# Low Friction CrN_MPP_/TiN_DCMS_ Multilayer Coatings

**DOI:** 10.1007/s11249-012-9922-y

**Published:** 2012-02-10

**Authors:** J. Paulitsch, C. Maringer, P. H. Mayrhofer

**Affiliations:** Department of Physical Metallurgy and Materials Testing, Montanuniversität Leoben, Franz-Josef-Str. 18, 8700 Leoben, Austria

**Keywords:** MPP, Ambient air, HIPIMS, BOD, Multilayer architecture, Superlattice

## Abstract

Transition metal nitrides like CrN and TiN are widely used in automotive applications due to their high hardness and wear resistance. Recently, we showed that a multilayer architecture of CrN and TiN, deposited using the hybrid—high power impulse magnetron sputtering (HIPIMS) and direct current magnetron sputtering (DCMS)—HIPIMS/DCMS deposition technique, results in coatings which indicate not only increased mechanical and tribological properties but also friction coefficients in the range of diamond-like-carbon coatings when tested at RT and ambient air conditions. The modulated pulsed power (MPP) deposition technique was used to replace the HIPIMS powered cathode within this study to allow for a higher deposition rate, which is based on the complex MPP pulse configuration. Our results on MPP/DCMS deposited CrN/TiN multilayer coatings indicate excellent mechanical and tribological properties, comparable to those obtained for HIPIMS/DCMS. Hardness values are around 25 GPa with wear rates in the range of 2 × 10^−16^ Nm/m^3^ and a coefficient of friction around 0.05 when preparing a superlattice structure. The low friction values can directly be correlated to the relative humidity in the ambient air during dry sliding testing. A minimum relative humidity of 13% is necessary to guarantee such low friction values, as confirmed by repeated tests, which are even obtained after vacuum annealing to 700 °C. Our results demonstrate that the co-sputtering of high metal ion sputtering techniques and conventional DC sputtering opens a new field of applications for CrN/TiN coatings as high wear resistance and low friction coatings.

## Introduction

Chromium and titanium nitride (CrN, TiN) thin films are the focus on many research activities as they are ideal candidates to study the interrelation between microstructure, morphology and resulting properties [1–4]. Physical vapour deposition (PVD) techniques like direct current magnetron sputtering (DCMS) or arc evaporation are mainly used to deposit such nitride hard coatings. The main difference between these two techniques is the ionization rate of the particles sputtered or evaporated from the target material (cathode), which is either below 10 or above 90%, respectively. Especially when prepared at lower temperatures and when using low bias potentials, DCMS coatings often suffer from under-dense morphologies due to the low ionization rate of sputtered target species and the moderate energy delivered to the growing film. On the other hand, coatings prepared by arc evaporation are in general characterized by very dense morphologies, due to the high ionization rate and available energies, but often exhibit a high density of macro particles (droplets) ejected from the cathode [5, 6]. By increasing the ion-to-neutral arrival ratio, e.g. by adjusting the magnetic field of DCMS or implementing filter technologies for arc deposition techniques improved film structures and thereby film properties can be obtained [2, 5–10]. On the other hand, high power impulse magnetron sputtering (HIPIMS) typically allows ionization rates above 40% of the sputtered particles due to the high power dissipation at the target [11–14], without the drawback of macro particle formation, and thereby can fill the gap between conventional DCMS and arc evaporation resulting in superior coatings properties [15–18]. However, the main drawback of HIPIMS is its generally low deposition rate. As an alternative to HIPIMS the modulated pulsed power (MPP) technique has been developed, exhibiting longer pulse durations, ranging from 400 to 3,000 μs, as well as multiple steps and micro pulses within a single pulse [19–21]. Due to this approach, low and high density discharges during one long pulse can be achieved resulting in typical maximum target power densities up to 0.5 kW/cm^2^ [21]. Such pulses result in a high ionization rate combined with deposition rates comparable to those of DCMS [20].

Although CrN and TiN are well-investigated coating materials and often serve as model systems for basic research concerning the chemistry–structure–properties relation, we recently could show that a multilayer arrangement of CrN/TiN, when prepared with HIPIMS in combination with DCMS, exhibits remarkably low ball-on-disc friction coefficients of ~0.1 and wear rates of ~3 × 10^−16^ m^3^/Nm [17, 18]. Here, we study the feasibility of MPP to obtain comparable results for multilayered coatings, where the Cr target is operated in MPP mode and the Ti target in DCMS mode. In agreement to previous results, also the prepared CrN_MPP_/TiN_DCMS_ exhibit friction coefficients of ~0.1, as well as the same dependence to the different ambient atmosphere used during testing.

## Experimental

All depositions were carried out using high purity metal Cr and Ti targets, in the size of 300 × 100 × 6 mm. The Ti cathode was powered using a continuous dc Pinnacle generator from Advanced Energy Inc. and the Cr cathode was powered by a Zpulser LLC MPP power supply. The substrates, steel disks (30 mm in diameter and 10 mm in height) and Si (001) strips (20 × 7 × 0.35 mm), are mounted on substrate holders which allow a frequent change of position to face the individual targets at a minimum distance of 16 cm necessary for the multilayer preparation. All depositions are performed without additional heating at a total pressure of 0.4 Pa in a mixed Ar/N_2_ glow discharge using a 1/1 flow rate ratio, and floated substrate potential. The multilayer coatings have been developed using a pulse repetition rate of 85 Hz and a powering of the MPP Cr cathode of 1.5 kW and a dc powering of the Ti cathode of 1 kW. To increase the adhesion of our coatings, an approximately 200 nm thick Cr interlayer was deposited, prepared by MPP using a repetition rate of 52 Hz and a cathode powering of 1.5 kW.

Prior to all depositions, the substrates are thermally cleaned at 500 °C within the vacuum chamber at a base pressure below 0.27 × 10^−5^ Pa, and both targets are sputter-cleaned with Ar ions. The substrates are ion etched in an Ar atmosphere using the Cr target in MPP mode and applying a substrate bias potential of −400 V for 20 min.

Fracture cross-section scanning electron microscopy (SEM) investigations of coated Si (001) substrates were conducted in a Zeiss EVO 50 microscope using an operating voltage of 20 kV. Detailed studies on morphology and film structure are evaluated using a Phillips CM 12 transmission electron microscope (TEM) operating at 120 kV. Structure and phase analyses of coated steel substrates are conducted by X-ray diffraction (XRD) in the Bragg–Brentano mode using a Siemens D500 equipped with a Cu *K*_α_ radiation source. Hardness (*H*) and modulus of indentation (*E*) of our coatings (evaluated on steel substrates) are obtained by nanoindentation with a Berkovich indenter using an ultra micro indentation system. The maximum loads are ranging from 10 to 35 mN to keep the indentation depth below 10% of the film thickness. The values for *H* and *E* were obtained from analysing the loading and unloading segments of the indentation curves after the Oliver–Pharr method [22]. Dry sliding tribological investigations are conducted at RT using a CSM ball-on-disk (BOD) tribometer equipped with an alumina ball (diameter of 6 mm) as counterpart. Alumina balls were used due to their increased thermal, chemical and wear resistance compared with our multilayer coating. A static normal load of 1 N, linear speed of 10 cm/s and sliding distances up to 2,000 m were used. Experiments are performed in ambient atmosphere (relative humidity ~25%) and in dry nitrogen (relative humidity ~1%). Our recent investigations showed that the gas used, either nitrogen, argon or synthetic air, for decreasing the humidity during BOD testing had no influence on the evaluated coefficient of friction [18]. Therefore, nitrogen was used as it allows us long time BOD studies due to a high amount available. The relative humidity was measured by a Testo 608-H_2_ hygrometer, with an error of ~2%. The sliding wear coefficients, of our multilayered coatings, are calculated after evaluating the wear track with a Wyko NT1000 optical profilometer. Biaxial stress-temperature measurements were carried out for coatings on Si (001) strips using the cantilever beam method and evaluated after the modified Stoney equation [23, 24].

## Results and Discussion

**Fig. 1:**
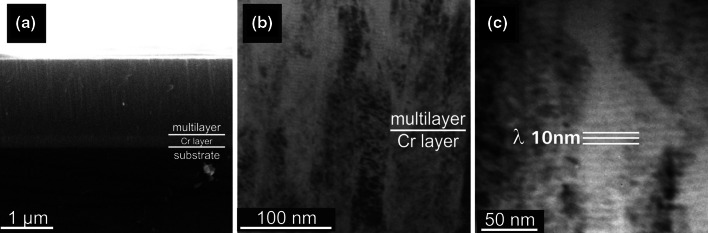
SEM, (**a**) and (**b**), and TEM (**c**) cross-sections of multilayered CrN_MPP_/TiN_DCMS_ coatings with a bilayer period of 10 nm. All coatings were deposited with a ~220 nm thick Cr interlayer to increase the coatings’ adhesion

SEM fracture cross-sections of our CrN_MPP_/TiN_DCMS_ multilayer coatings deposited with a repetition frequency of 85 Hz, Fig. 1a and b, indicate dense fibrous structures, smooth surfaces and an approximately 220 nm thick Cr interlayer. The surface roughness values *R*_a_ of our coatings, evaluated with an optical profilometer, are around 6 nm. The cross-sectional TEM investigations, Fig. 1c, demonstrate the dense columnar structure with alternating CrN and TiN layers with sharp interfaces. The thicknesses of the individual TiN layers are around 3.5 nm and that for the CrN layers are ~6.5 nm, hence our multilayers have a bilayer period of 10 nm, which is corresponding to the previously reported low friction CrN_HIPIMS_/TiN_DCMS_ coatings [17, 18]. These multilayer coatings, prepared by the hybrid HIPIMS/DCMS technology, exhibit friction coefficients of ~0.07 combined with an excellent wear resistance of ~3 × 10^−16^ m^3^/Nm when tested at 20 °C using dry sliding against an alumina ball in ambient air with a relative humidity of 25 ± 2%.

**Fig. 2:**
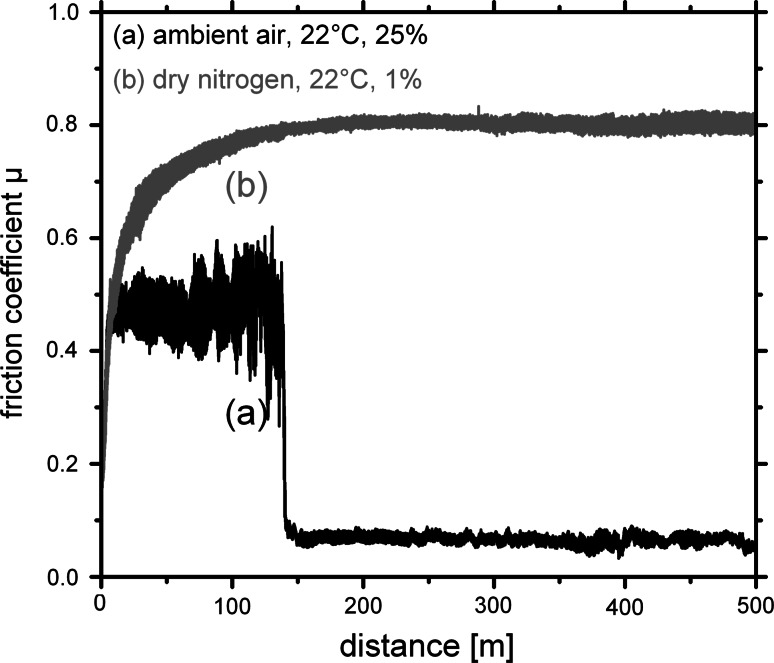
Friction coefficient μ of multilayered CrN_MPP_/TiN_DCMS_ coatings evaluated by dry sliding BOD tests equipped with an Al_2_O_3_ ball at ambient air conditions with a relative humidity of ~25% and in dry nitrogen at ~1%

**Fig. 3:**
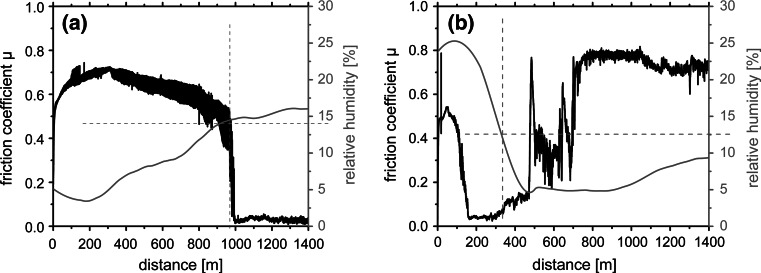
Friction coefficient μ curves of multilayered CrN_MPP_/TiN_DCMS_ coatings as a function of increasing and decreasing relative humidity during BOD testing, (**a**) and (**b**), respectively

**Fig. 4:**
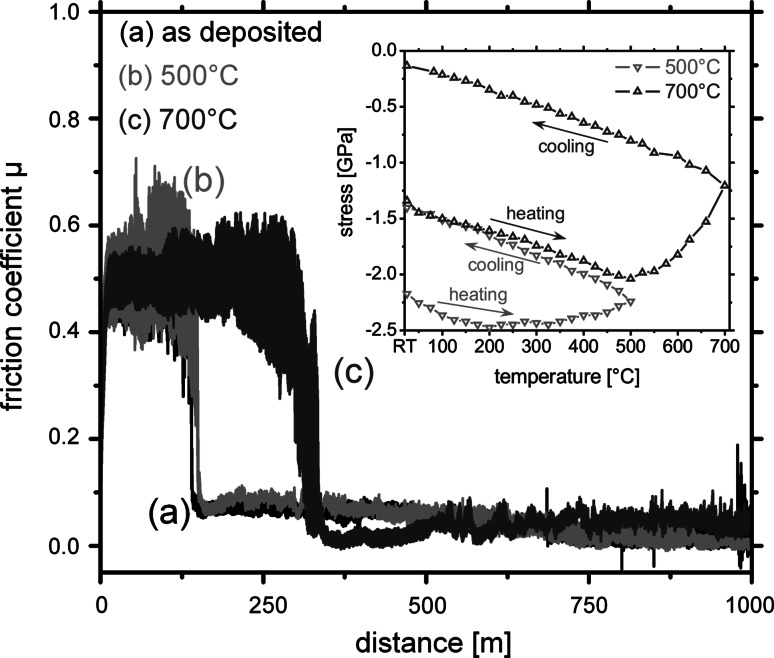
Friction coefficient μ of multilayered CrN_MPP_/TiN_DCMS_ coatings in the as-deposited state and after vacuum annealing at 500 and 700 °C, (**a**) to (**c**), respectively. The inset indicates the in situ evaluated biaxial stress–temperature curves during vacuum annealing at 500 and 700 °C

The CrN_MPP_/TiN_DCMS_ coatings, prepared with modulated pulse power and dc magnetron sputtering, indicate comparable friction behaviour at ambient air, see Fig. 2. After a short running-in period friction coefficients μ below 0.05 can be obtained. Corresponding to the previous studies, also these coatings indicate decreased volume reduction during BOD testing resulting in high wear resistance with wear rate values as low as ~2 × 10^−16^ m^3^/Nm, suggesting that when using MPP instead of HIPIMS during the combination with DCMS, comparable coatings can be obtained. Performing the BOD test in dry nitrogen with a relative humidity of ~1% shows the expected, based on the previous studies presented in [18], increase in coefficient of friction to values in the range of 0.8 for our multilayer coatings. The experimental setup for the measurements in defined atmospheres is described in more detail in Ref. [18]. For obtaining the minimum humidity necessary to have friction coefficients below 0.1 during dry sliding tests, the relative humidity was adjusted by mixing ambient air and dry nitrogen. Figure 3a shows that by increasing the relative humidity above 13% the friction coefficient drops from ~0.7 to values below 0.05. Correspondingly, when decreasing the relative humidity, the friction coefficients start to increase from ~0.05 to 0.7 at a critical value of ~13%, see Fig. 3b. This effect, of changing friction coefficient from high to low values with varying the humidity, can be obtained in a cyclic manner within one testing run. Therefore, we can assume that due to the humidity in the ambient air a tribo-layer is formed resulting in the low friction values. If the supply of lubricant is stopped, in our case due to the reduction of humidity during testing by introducing nitrogen, no lubricant film can be formed between the coating and the alumina ball resulting in an increase in the coefficient of friction. Figure 3b also indicates a certain drop of the μ value at a measurement distance of around 500–600 m. These decreased values are due to coating fatigue and resulting abrasive coating material within the wear track, which show bearing-like behaviour before being polished-in or removed from the wear track. To study the influence of the residual coating stresses on the friction coefficient, the coatings were annealed in vacuum to 500 and 700 °C, which results in a reduction of their biaxial stresses from the as deposited −2.2 GPa to −1.4 and −0.2 GPa, respectively, see the inset in Fig. 4. The BOD tests after these thermal treatments exhibit friction curves comparable to the as-deposited state with a steady-state friction coefficient μ around 0.05, see Fig. 4. Consequently, the friction coefficient of our multilayer coatings is independent of their residual stresses, in the tested regime. The observed difference in the running-in period is depending on the radius used during BOD testing when plotting μ vs. distance indicating a necessary asperity polishing and scratching of the coating surface to obtain the low-friction effect. These scratching-in depths are close to being constant for all measurements at around 100 nm and indicate to be independent to the sliding velocity used.

**Fig. 5:**
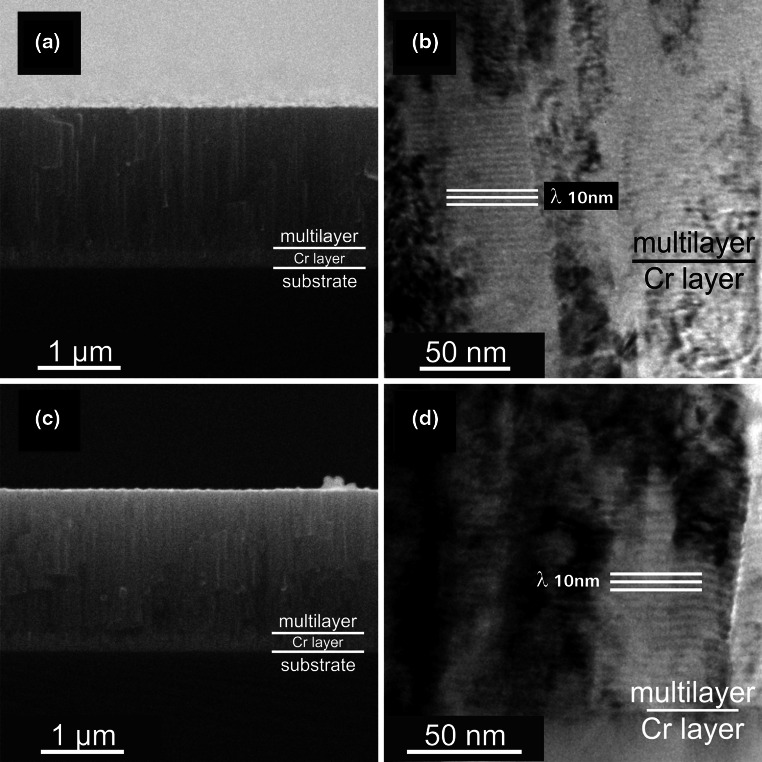
SEM, (**a**) and (**b**), and TEM, (**c**) and (**d**), cross-sections of our multilayered CrN_MPP_/TiN_DCMS_ after vacuum annealing at 500 and 700 °C, respectively

**Fig. 6:**
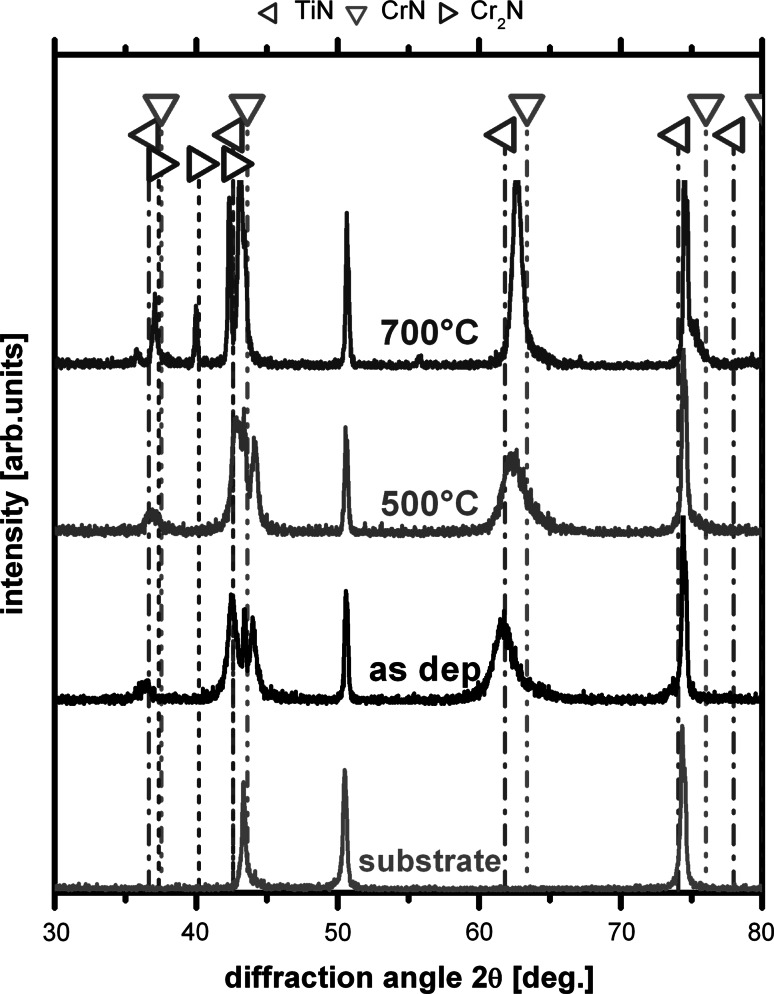
XRD analysis of the as-deposited and vacuum-annealed CrN_MPP_/TiN_DCMS_ multilayer coatings with a bilayer period of 10 nm

**Fig. 7:**
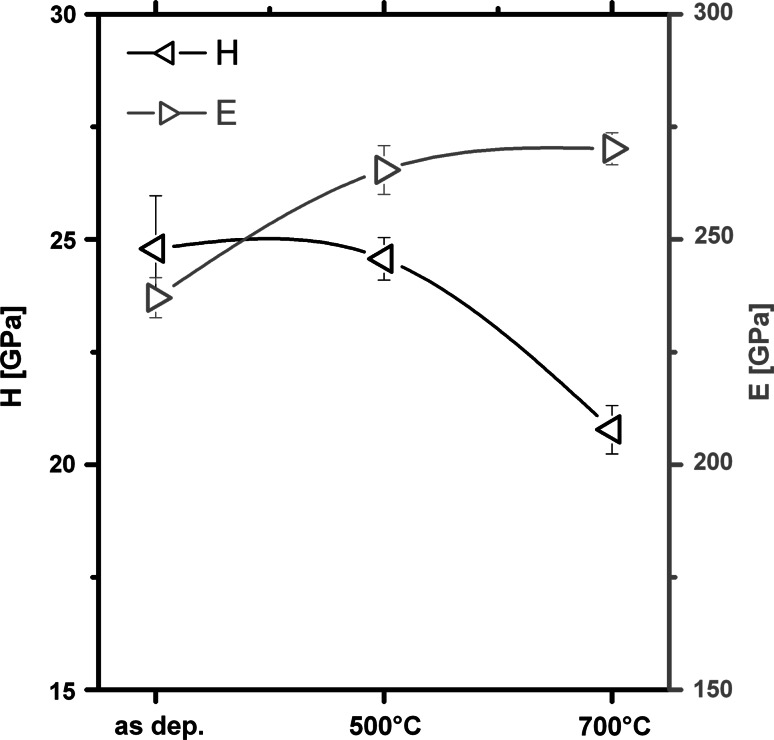
Indentation hardness (*H*) and modulus of indentation (*E*) of the as-deposited and vacuum-annealed CrN_MPP_/TiN_DCMS_ multilayer coatings with a bilayer period of 10 nm

SEM and TEM investigations of the coatings after the annealing treatment to 500 °C, Fig. 5a and b, and 700 °C, Fig. 5c and d, indicate no significant change in the overall morphology and bilayer arrangement and period. Nevertheless, XRD studies clearly show that the orientation and crystal feature size of the coatings increases when annealed to 700 °C, while annealing to 500 °C exhibits no significant change with respect to the as-deposited state, see Fig. 6. Annealing to 700 °C results in a change of the multilayer coatings from single-phase cubic structured with preferred 111 orientation to more randomly oriented cubic dominated structure with a small fraction of hexagonal Cr_2_N content, see Fig. 6. Consequently, the hardness of the coatings is constant at 25 ±2 GPa for annealing temperatures to 500 °C but decreases to 20 ± 2 GPa due to the annealing at 700 °C, see Fig. 7. The slightly increased indentation modulus due to annealing at 500 and 700 °C, which is an indication for a reduced defect density and increased crystalline feature size, confirms the XRD results. These studies suggest that the friction coefficient of the multilayer coatings, which is almost independent of the annealing treatment to 700 °C, is dominated by the bilayer arrangement and period of the individual MPP-CrN and DCMS-TiN layers, which are both also almost independent of the annealing treatment to 700 °C.

## Summary and Conclusion

CrN/TiN multilayer coatings with a bilayer thickness of 10 nm were deposited using the hybrid MPP/DCMS technique. These films were investigated for their tribological properties in a sliding contact in ambient air and by varying the atmospheric conditions by introducing dry nitrogen. The measurements showed that coatings with high hardness values, around 25 GPa, and low wear rates, in the range of 2 × 10^−16^ Nm/m^3^, can be developed. Furthermore, our coatings indicate a coefficient of friction μ below 0.1, which is comparable to diamond-like carbon films. By decreasing the relative humidity during testing we demonstrate that such steady-state friction values in the range of 0.05–0.1 can only be achieved if the relative humidity exceeds 13%. Investigations after vacuum annealing to 500 and 700 °C indicate a reduction of residual film stresses from around −2.2 GPa, in the as-deposited state, to −1.4 and −0.2 GPa, respectively. Nevertheless, such heat treatments are not affecting the dry sliding behaviour, as still steady-state μ values of ~0.05 can be observed. As the multilayer architecture of our coatings is not significantly influenced by these vacuum annealing steps, we can conclude that the low friction coefficient mainly depends on the coatings’ architecture and bilayer period.

Our results suggests that CrN/TiN superlattice coatings, prepared by combining high and low ionizing deposition techniques, can be used for tribological applications (especially in at least 13% humid atmospheres) where high hardness and wear stability and low friction coefficients are essential.

## References

[1] Hultman L, Sundgren JE, Markert LC, Greene JE (1989). Ar and excess N incorporation in epitaxial TiN films grown by reactive bias sputtering in mixed Ar/N-2 and pure N-2 discharges. J. Vac. Sci. Technol. A.

[2] Petrov I, Barna PB, Hultman L, Greene JE (2003). Microstructural evolution during film growth. J. Vac. Sci. Technol. A.

[3] Petrov I, Hultman L, Sundgren JE, Greene JE (1992). Polycrystalline TiN films deposited by reactive bias magnetron sputtering—effects of ion-bombardment on resputtering rates, film composition, and microstructure. Vac. Sci. Technol. A.

[4] Sundgren JE (1985). Structure and properties of TiN coatings. Thin Solid Films.

[5] Mayrhofer PH, Geier M, Locker C, Chen L (2009). Influence of deposition conditions on texture development and mechanical properties of TiN coatings. Int. J. Mater. Res..

[6] Håkansson G, Hultman L, Sundgren JE, Greene JE, Münz WD (1991). Microstructures of TiN films grown by various physical vapour deposition techniques. Surf. Coat. Technol..

[7] Greene JE, Barnett SA (1982). Ion surface interactions during vapor-phase crystal-growth by sputtering, MBE, and plasma-enhanced CVD—applications to semiconductors. J. Vac. Sci. Technol..

[8] Petrov I, Hultman L, Helmersson U, Sundgren JE, Greene JE (1989). Microstructure modification of TiN by ion bombardment during reactive sputter deposition. Thin Solid Films.

[9] Hultman L, Münz WD, Musil J, Kadlec S, Petrov I, Greene JE (1991). Low-energy (-100 Ev) ion irradiation during growth of TiN deposited by reactive magnetron sputtering—effects of ion flux on film microstructure. J. Vac. Sci. Technol. A.

[10] Mayrhofer PH, Kunc F, Musil J, Mitterer C (2002). A comparative study on reactive and non-reactive unbalanced magnetron sputter deposition of TiN coatings. Thin Solid Films.

[11] Ehiasarian AP, Hovsepian PE, Hultman L, Helmersson U (2004). Comparison of microstructure and mechanical properties of chromium nitride-based coatings deposited by high power impulse magnetron sputtering and by the combined steered cathodic arc/unbalanced magnetron technique. Thin Solid Films.

[12] Ehiasarian AP, Münz WD, Hultman L, Helmersson U, Petrov I (2003). High power pulsed magnetron sputtered CrNx films. Surf. Coat. Technol..

[13] Ehiasarian AP, New R, Münz WD, Hultman L, Helmersson U, Kouznetsov V (2002). Influence of high power densities on the composition of pulsed magnetron plasmas. Vacuum.

[14] Kouznetsov V, Macák K, Schneider JM, Helmersson U, Petrov I (1999). A novel pulsed magnetron sputter technique utilizing very high target power densities. Surf. Coat. Technol..

[15] Paulitsch, J., Mayrhofer, P.H., Mitterer, C., Münz, W.D., Schenkel, M.: Mechanical and tribological properties of CrN coatings deposited by a simultaneously HIPIMS/UBM sputtering process. In: Proceedings, 50th Annual Technical Conference—Society of Vacuum Coaters, pp. 150–154 (2007)

[16] Paulitsch J, Mayrhofer PH, Münz WD, Schenkel M (2008). Structure and mechanical properties of CrN/TiN multilayer coatings prepared by a combined HIPIMS/UBMS deposition technique. Thin Solid Films.

[17] Paulitsch J, Schenkel M, Zufraß T, Mayrhofer PH, Münz WD (2010). Structure and properties of high power impulse magnetron sputtering and DC magnetron sputtering CrN and TiN films deposited in an industrial scale unit. Thin Solid Films.

[18] Paulitsch J, Schenkel M, Schintlmeister A, Hutter H, Mayrhofer PH (2010). Low friction CrN/TiN multilayer coatings prepared by a hybrid high power impulse magnetron sputtering/DC magnetron sputtering deposition technique. Thin Solid Films.

[19] Lin J, Moore JJ, Sproul WD, Mishra B, Rees JA, Wu Z, Chistyakov R, Abraham B (2009). Ion energy and mass distributions of the plasma during modulated pulse power magnetron sputtering. Surf. Coat. Technol..

[20] Lin J, Moore JJ, Sproul WD, Mishra B, Wu Z (2009). Modulated pulse power sputtered chromium coatings. Thin Solid Films.

[21] Chistyakov, R., Abraham, B.: Modulated pulse power technology and deposition for protective and tribological coatings. In: Proceedings, 50th Annual Technical Conference—Society of Vacuum Coaters, pp. 139–143 (2007)

[22] Oliver WC, Pharr GM (1992). Improved technique for determining hardness and elastic modulus using load and displacement sensing indentation experiments. J. Mater. Res..

[23] Wilcock JD, Campbell DS (1969). A sensitive bending beam apparatus for measuring the stress in evaporated thin films. Thin Solid Films.

[24] Mayrhofer PH, Mitterer C (2000). High-temperature properties of nanocomposite TiBxNy and TiBxCy coatings. Surf. Coat. Technol..

